# Supplementary material for: Allocating capital-associated CO2 emissions along the full lifespan of capital investments helps diffuse emission responsibility
Source: Nat Commun. 2023 May 11;14:2727. doi: 10.1038/s41467-023-38358-z (PMC10173932; doi:10.1038/s41467-023-38358-z)
Supplement: Supplementary file 1 — Supplementary Information [file 41467_2023_38358_MOESM1_ESM.pdf]

Supplementary Information (SI)

for

Allocating capital-associated CO_2 emissions along full lifespan of capital investments helps re-assessing emission responsibilities

Quanliang Ye, Maarten S. Krol, Yuli Shan*, Joep F. Schyns, Markus Berger, Klaus Hubacek*

*Corresponding authors:

Dr. Yuli Shan
Email: y.shan@bham.ac.uk
ORCID: 0000-0002-5215-8657

Prof. Klaus Hubacek
Email: k.hubacek@rug.nl
ORCID: 0000-0003-2561-6090

SI Contents

1. Comparison of existing methods for capital-oriented environmental pressure assessments 3
Table S1. Comparison of existing capital-oriented environmental pressure assessment methods. 3
2. Capital investment in China 4
Figure S1. Capital investment (left y-axis) and the share of gross fixed capital formation (GFCF) in national value-added (right y-axis) of China during the period 1995-2017.......... 4
3. Carbon emissions under capital scenarios.......... 5
Figure S2. Regional carbon emissions (in Gt) for the year 2017, and year 2030 under the three capital investment scenarios. 5
Figure S3. Sectoral contributions to the regional carbon emission changes (in Mt) in 2030 under the three capital investment scenarios. 6
4. Annual profile of capital-considered carbon emissions 7
Figure S4. Annual profiles of national carbon emissions (in Gt) with the re-allocation of capital-related emissions (F^K) during the period 2018-2030. 7
5. Data Sources 8
5.1 Capital data.......... 8
Table S2. Categories of sectors of newly increased fixed assets (NIFA) recorded by the National Bureau of Statistics of China. 8
Table S3. Categories of 37 capital investing sectors recorded in the WorldKLEMS. 10
5.2 MRIO-related data.......... 11
Table S4. Gross regional product by expenditure approach for the year 2017. 11
5.3 Carbon emission inventory 12
6. Total investment in fixed assets (TIFA) V.S. newly increased fixed assets (NIFA).......... 13
Figure S5. Relationship between Chinese statistical capital data. 13
7. The Perpetual Inventory Method (PIM) 15
8. Constructing China's inter-provincial MRIO table series (1995-2017).......... 16
Table S5. Provincial value added in 2012 recorded in the National Bureau of Statistics of China (NBSC), MRIO tables from Liu et al. and Wang, respectively.. 16
Table S6. List of the 42 sectors in China's inter-provincial MRIO table time series. 17
9. Constructing capital consumption time series 19
10. Summary of the 'business-as-usual' (BAU) scenario and the two capital investment scenarios 20
Table S7. Summary of the 'business-as-usual' (BAU) scenario and the two capital investment scenarios. 20
11. Major parameters in the three capital investment scenarios. 21
Table S8. Major parameters in the three capital investment scenarios. 21
12. Applying energy mix changes in MRIO tables 22
Figure S6. Diagram to apply energy mix changes in MRIO tables. 22
13. Relationships between sectorial capital investment and final consumption 23
Figure S7. Trends in capital investment by, and final consumption of electricity/water production and supply sector as well as transportation service sector. 23
14. Changes in per-capital CO_2 emissions 24
Table S9. Changes in regional per-capita PBEs and CBEs for the year 2017 with and without the re-allocation of F^K. 24
15. The logic of temporal allocation of capital-associated CO_2 emissions to production-based emissions.......... 25
SI References.......... 26

1. Comparison of existing methods for capital-oriented environmental pressure assessments

A common idea of considering capital activities into environmental pressure assessment is endogenizing capital transactions into input-output tables[1]. There have been two prominent methods for capital endogenization in the early studies, the augmentation method and the flow matrix method.[1] The augmentation method incorporates the gross fixed capital formation (GFCF, a column vector of final demand) and consumption of fixed capital (CFC, a row vector of value added) as an additional column and row into the intermediate input matrix. This artificially created sector is assumed to produce one homogeneous commodity 'capital', which is produced using input according to GFCF and consumed by other sectors according to CFC. The flow matrix method disaggregates capital consumption data by producing assets and consuming sectors. This disaggregation results in a capital flow matrix which could be combined with the conventional intermediate input matrix to form a new flow matrix including both monetary inputs and capital consumption. Based on previous attempts, the augmentation method, although being easier to implement, led to a systematic distortion in the calculated factor multipliers[1,2]; whereas the flow matrix method could provide more sensible results but the main drawback is its relatively high data requirements[3,4,5].

However, both the augmentation method and the flow matrix method don't consider the intertemporal feature of capital assets (Table S1)[3]. In another word, the fact that capital assets used for year *n*'s production are from different time cohorts—produced based on different production recipes, trade networks, and environmental intensities—has been neglected in previous methods. This neglection has been found to result in an approximate 30% underestimation in capital-related GHG emissions[4]. To tackle this intertemporal feature of capital in environmental pressure assessments, Ye et al.[3] developed a novel method that broke down capital consumption in a certain year into different investment years in the past, and address the temporal dynamic of capital investment and consumption in conventional input-output model. The limitation of Ye et al.[3] is the lack of understanding how the existing capital assets will sever future production and consumption under different capital investment alternatives.

Table S1. Comparison of existing capital-oriented environmental pressure assessment methods.

	Literature				
	Lenzen and Treloar [1]	Södersten, et al. [4,5,6]	Chen, et al. [2]	Ye, et al. [3]	This study
Endogenizing method					
The augmentation method	√		√		
The flow matrix method	√	√			
Dynamic capital consumption method				√	√
Whether clearly distinguishing capital activities from investment, formation, use					√
Whether considering temporal dynamic of capital assets			√ (without time-series analysis)	√	√
Whether exploring future influences of existing capital					√

2. Capital investment in China

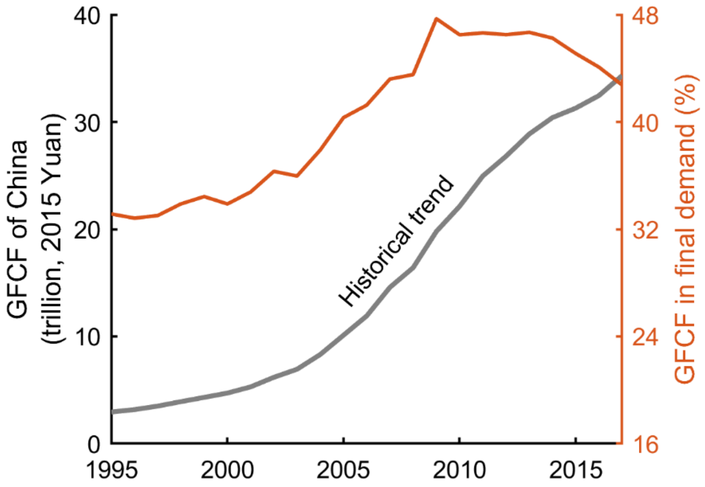

Figure S1. Capital investment (left y-axis) and the share of gross fixed capital formation (GFCF) in national value-added (right y-axis) of China during the period 1995-2017.

3. Carbon emissions under capital scenarios

National PBEs and CBEs would substantially increase under the BAU and KES scenarios compared with those in 2017 (Figure 3a in the main text), while under the KLC scenario, only a slight growth (less than 2%) is observed for them and potential decreases could also be expected in some certain regions (e.g., the Beijing-Tianjin, and the Southwest, see Figure S2).

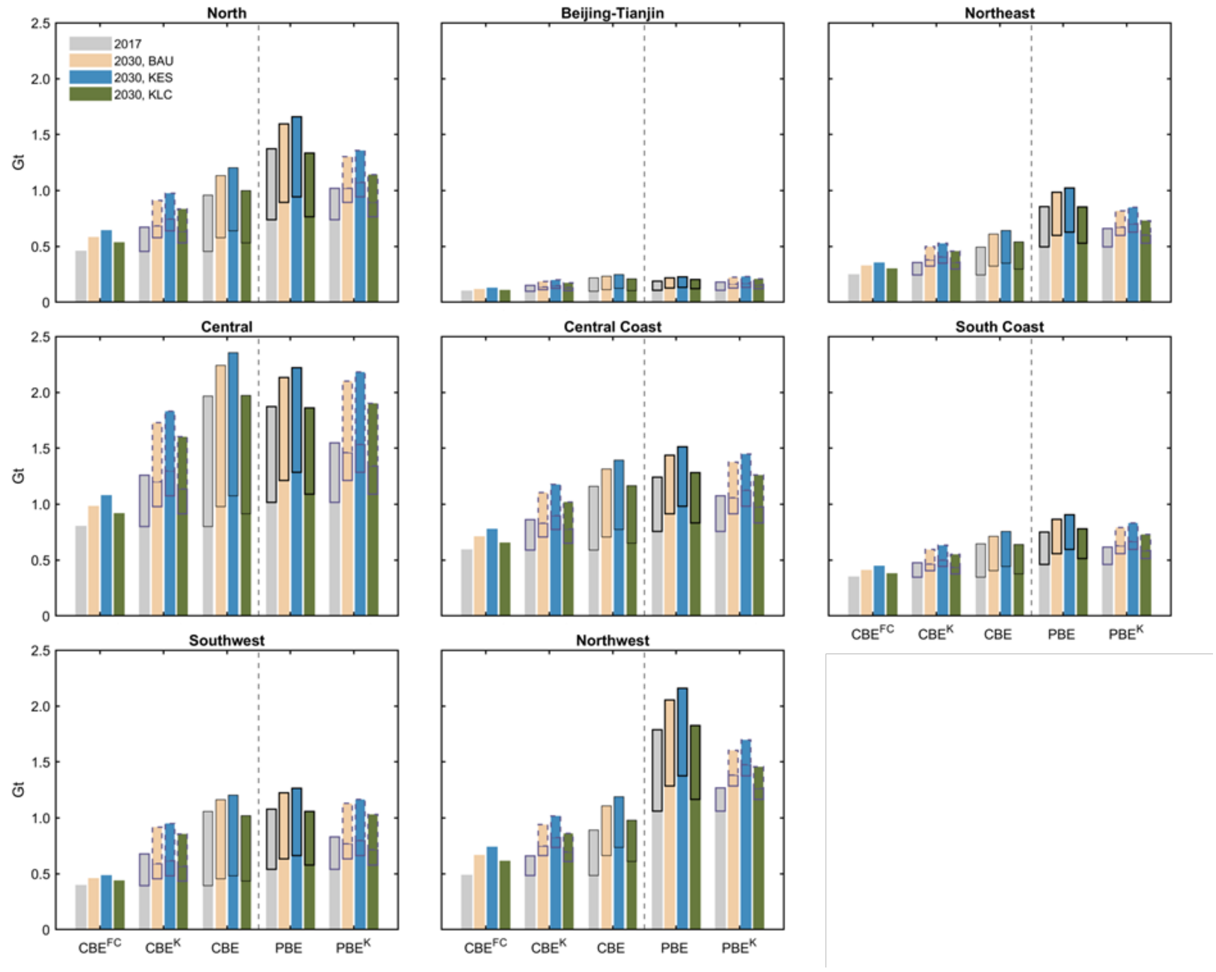

Figure S2. Regional carbon emissions (in Gt) for the year 2017, and year 2030 under the three capital investment scenarios. In each panel, capital-related carbon emissions (F^K) are disaggregated into those occurred in the period of 1995−2017 (with solid edge line) and those would occur in the period of 2018−2030 (with dashed edge line).

From a production perspective, the national PBE in 2030 (Figure 3b in the main text) would increase by 15% under the BAU scenario from the base-year level, and by 20% under the KES scenario because more investment will be made in infrastructure for economic growth and social well-being improvement. The main growth in national PBEs under the BAU and KLC would be observed in transportation services due to the increase of its final consumption, whereas offset by carbon emissions from electricity generation given the efficiency improvement of production and energy use (Figure S3). From the consumption perspective, similar growth rates would also be found in national consumption-based carbon emissions of final consumption and final demand, showing the largest changes under the KES scenario (by 35% and 22%, respectively) whereas least changes under the KLC scenario (by 15% and 1.8%, respectively). Moreover, at the regional level, the relative changes in regional consumption-based carbon emissions of final demand are larger in less developed regions such as the Northeast and the Northwest (+9–34%), mainly due to the growth of electricity generation, construction, and transportation services. In comparison, the changes in consumption-based carbon

emissions of final demand in highly developed regions like Beijing-Tianjin, the Central Coast, and the South Coast would be in the range of -4–+20%.

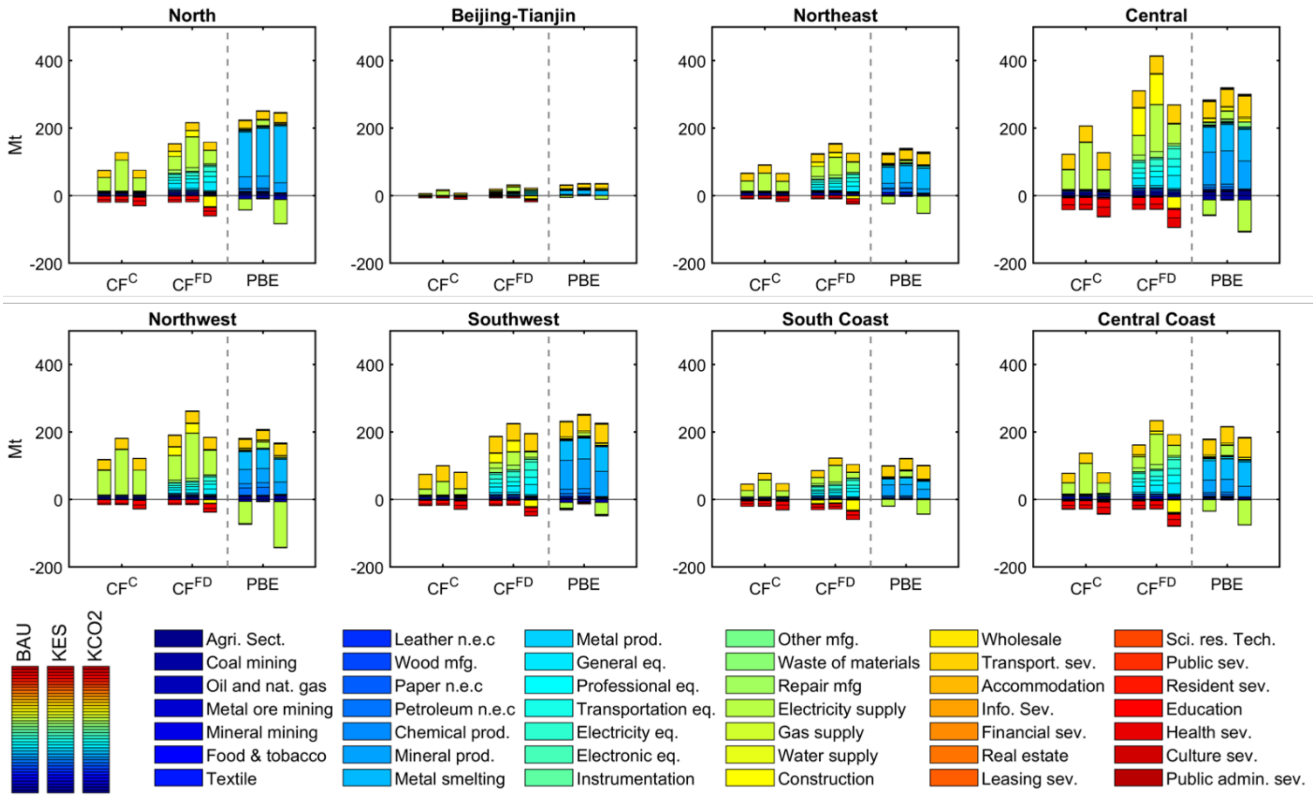

Figure S3. Sectoral contributions to the regional carbon emission changes (in Mt) in 2030 under the three capital investment scenarios. *CF^C* and *CF^{FD}* represent the carbon footprint of final consumption and final demand, respectively, by conventional consumption-base accounting. PBE represents the production-based carbon emissions, excluding the carbon emissions embodied in international exports.

4. Annual profile of capital-considered carbon emissions

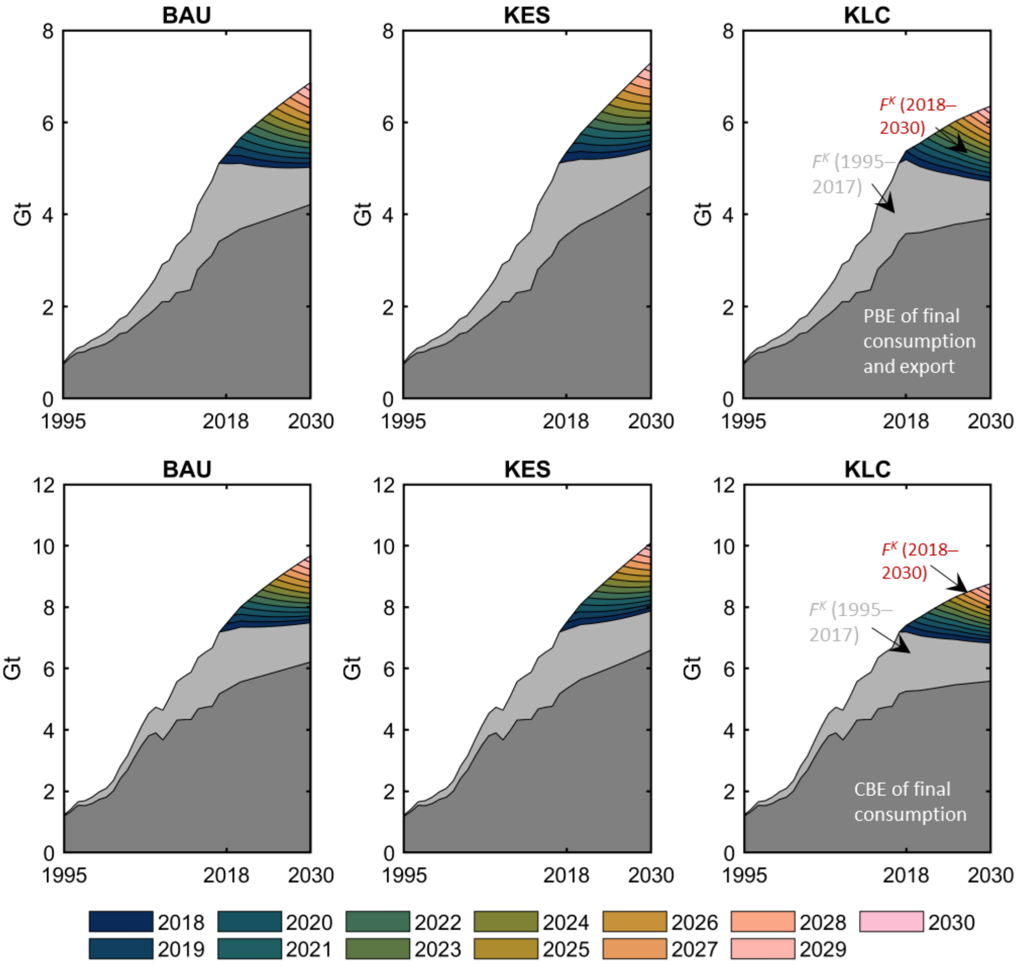

Figure S4. Annual profiles of national carbon emissions (in Gt) with the re-allocation of capital-related emissions (F^K) during the period 2018-2030.

The re-allocated F^K is further disaggregated into those occurred in the period of 1995-2017 (in grey color tone) and those would occur in the period of 2018-2030 (in bright color tones).

5. Data Sources

5.1 Capital data

The major capital data we use in this study include total investment in fixed assets (TIFA) by sector and by province, newly increased fixed assets (NIFA) by sector and by province, and depreciation rates by asset and by capital consuming sectors. TIFA and NIFA are mainly collected from the statistical database of the National Bureau of Statistics of China (NBSC)[7], and the Statistical Yearbook of the Chinese Investment in Fixed Assets[8]. Official NIFA are distinguished as rural NIFA and urban NIFA by 19 major economic sectors (e.g., "Agriculture, Forestry, Animal Husbandry and Fishery" or "Construction", see Table S2). Particularly, urban NIFA are also recorded by 40 specific industrial sectors (e.g., "Food Manufacturing" or "Electricity, Heat Production and Supply", see Table S2). However, the industrial classifications of the official data are inconsistent overt time. That is, before 2002, sector "*Hotels and Catering Services*" was aggregated into sector "*Wholesale and Retail Trades*"; sector "*Education*" was aggregated into sector "*Cultural, Sports, and Entertainment Services*"; while sectors "*Information Transmission, Computer Services and Software*", "*Leasing and Business Services*", "*Services to Households and Other Services*" and "*Public Administration and Social Organization Services*" were aggregated as "*Public Services*". To ensure the consistency of sectoral classification during the study period, we re-allocate these aggregated sectors' capital investment into each sector based on their shares in the capital investment in the year 2003. Furthermore, rural NIFA of major industry sectors, i.e., "*Mining and Quarrying Industry*", "*Manufacturing Industry*", and "*Production and Supply of Electricity, Gas and Water*", are disaggregated into 40 specific industrial sectors based on their shares in urban NIFA in each province.

Table S2. Categories of sectors of newly increased fixed assets (NIFA) recorded by the National Bureau of Statistics of China.

**Rural NIFA**	**Urban NIFA**
Agriculture, Forestry, Animal Husbandry and Fishery	Agriculture, Forestry, Animal Husbandry and Fishery
Mining and Quarrying Industry	Mining and Quarrying Industry
Manufacturing Industry	Mining and Washing of Coal
Production and Supply of Electricity, Gas and Water	Petroleum and Natural Gas
Construction	Mining and Processing of Ferrous Metal Ores
Wholesale and Retail Trades	Mining and Processing of Non-Ferrous Metal Ores
Transport, Storage and Post	Minging and Processing of Nonmetal Ores
Hotels and Catering Services	Mining Supportive Activities
Information Transmission, Computer Services and Software	Minging of Other Ores
Financial Intermediation	Manufacturing Industry
Real Estate Services	Processing of Food from Agricultural Products
Leasing and Business Services	Manufacture of Foods
Scientific Research, Technical Service and Geologic Prospecting	Manufacture of Beverages
Management of Water Conservancy, Environment and Public Facilities	Manufacture of Tobacco
Services to Households and Other Services	Manufacture of Textile
Education	Manufacture of Textile Wearing Apparel, Footware and caps

Healthy Services	Manufacture of Leather, Fur, Feather and Related Products
Cultural, Sports, and Entertainment Services	Processing of Timber, Manufacture of Wood, Bamboo, Rattan, Palm and Straw Products
Public Administration and Social Organization Services	Manufacture of Furniture
	Manufacture of Paper and Paper Products
	Printing, Reproduction of Recording Media
	Manufacture of Articles For Culture, Education and Sports Activities
	Processing of Petroleum, Coking, Processing of Nuclear Fuel
	Manufacture of Raw Chemical Materials and Chemical Products
	Manufacture of Medicines
	Manufacture of Chemical Fibers
	Manufacture of Rubber and Plastics Products
	Manufacture of Non-metallic Mineral Products
	Smelting and Pressing of Ferrous Metals
	Smelting and Pressing of Non-ferrous Metals
	Manufacture of Metal Products
	Manufacture of General Purpose Machinery
	Manufacture of Special Purpose Machinery
	Manufacture of Transport Equipment
	Manufacture of Electrical Machinery and Equipment
	Manufacture of Communication Equipment, Computers and Other Electronic Equipment
	Manufacture of Measuring Instruments and Machinery for Cultural Activity and Office Work
	Manufacture of Artwork and Other Manufacturing
	Recycling and Disposal of Waste
	Metal Product Machinery and Equipment Repair Industry
	Production and Supply of Electricity, Gas and Water
	Production and Supply of Electric Power and Heat Power
	Production and Supply of Gas
	Production and Supply of water
	Construction
	Wholesale and Retail Trades
	Transport, Storage and Post
	Hotels and Catering Services
	Information Transmission, Computer Services and Software
	Financial Intermediation
	Real Estate Services
	Leasing and Business Services
	Scientific Research, Technical Service and Geologic Prospecting

	Management of Water Conservancy, Environment and Public Facilities
	Services to Households and Other Services
	Education
	Healthy Services
	Cultural, Sports, and Entertainment Services
	Public Administration and Social Organization Services

Table S3. Categories of 37 capital investing sectors recorded in the WorldKLEMS [9].

Sector Number	Full Name
1	Agriculture, forestry, animal husbandry & fishery
2	Coal mining
3	Oil & gas excavation
4	Metal mining
5	Non-metallic minerals mining
6	Food and kindred products
7	Tobacco products
8	Textile mill products
9	Apparel and other textile products
10	Leather and leather products
11	Saw mill products, furniture, fixtures
12	Paper products, printing & publishing
13	Petroleum and coal products
14	Chemicals and allied products
15	Rubber and plastics products
16	Stone, clay, and glass products
17	Primary & fabricated metal industries
18	Metal products (excluding rolling products)
19	Industrial machinery and equipment
20	Electric equipment
21	Electronic and telecommunication equipment
22	Instruments and office equipment
23	Motor vehicles & other transportation equipment
24	Miscellaneous manufacturing industries
25	Power, steam, gas and tap water supply
26	Construction
27	Wholesale and retail trades
28	Hotels and restaurants
29	Transport, storage & post services
30	Information & computer services
31	Financial Intermediations
32	Real estate services
33	Leasing, technical, science & business services
34	Government, public administration, and political and social organizations, etc.
35	Education
36	Healthcare and social security services
37	Cultural, sports, entertainment services; residential and other services

5.2 MRIO-related data

According to the data needed to construct China's inter-provincial MRIO table series, the associated data sources include: the current best available MRIO tables collected for the year 2007 [10], 2010 [11], 2012 [12], 2015 and 2017 from CEADs [13], and 1995-2016 from Wang [14]; product-specific per-capita expenditures of rural and urban population in each province from China Statistical Yearbooks [15]; rural and urban population of each province from China Statistical Yearbook [15], and China Rural Statistical Yearbook [16]; gross final expenditures, GFCF, and stock changes of each province from China Statistical Yearbook [15], see Table S4 for associated data in 2017; product-specific export data from China Statistical Yearbook [15], China Trade And External Economic Statistical Yearbook [17], and Market Statistical Yearbook Of China [18].

Table S4. Gross regional product by expenditure approach for the year 2017. Unit: 10^8 Yuan.

Region	Province	Final expenditure			GFCF	Stock changes
		Rural	Urban	Government		
Beijing-Tianjin	Beijing	768	10724	5351	10375	768
Beijing-Tianjin	Tianjin	638	5441	2346	10138	638
North	Hebei	3486	8425	4144	19035	3486
Northwest	Shanxi	1801	4894	2062	6700	1801
Northwest	Inner Mongolia	1375	4661	2428	10392	1375
Northeast	Liaoning	1927	8948	2903	9639	1927
Northeast	Jilin	1089	3010	1701	10014	1089
Northeast	Heilongjiang	1753	5402	2967	9651	1753
Central Coast	Shanghai	756	12214	4581	11507	756
Central Coast	Jiangsu	6810	25083	11128	36417	6810
Central Coast	Zhejiang	4334	14702	6443	21862	4334
Central	Anhui	2827	7843	2829	13569	2827
South Coast	Fujian	2491	7617	3043	17638	2491
Central	Jiangxi	2553	5413	2258	9738	2553
North	Shandong	7431	20854	6901	34704	7431
Central	Henan	4978	12052	6100	30415	4978
Central	Hubei	3026	9729	4417	20587	3026
Central	Hunan	3645	9639	4792	17160	3645
South Coast	Guangdong	5385	28712	11032	38391	5385
Southwest	Guangxi	2325	5522	2658	9035	2325
South Coast	Hainan	465	1465	852	2809	465
Southwest	Chongqing	1181	5838	2271	9907	1181
Southwest	Sichuan	5323	9518	4525	17689	5323
Southwest	Guizhou	1935	3897	1674	9086	1935
Southwest	Yunnan	2335	5265	2906	14826	2335
Southwest	Shaanxi	1650	5419	2607	14144	1650
Central	Gansu	1054	2664	1430	3557	1054
Northwest	Qinghai	337	737	742	3918	337
Northwest	Ningxia	348	1081	685	3836	348
Northwest	Xinjiang	1185	2867	3220	10695	1185

5.3 Carbon emission inventory

Carbon emissions by sector of 30 regions during the period of 1997-2017 are collected from emission inventories complied by CEADs [19-21]. The CEADs' carbon emission inventories are constructed in a resolution of 45 sectors, as well as household emissions of rural and urban population. We aggregate the 45-sectorial emission data into the resolution of 42 MRIO-sectors. Moreover, there are still two-year carbon emission data missing. We assume the sectorial carbon emission intensities of sectors in 1995 and 1996 are equal to those in 1997.

6. Total investment in fixed assets (TIFA) V.S. newly increased fixed assets (NIFA)

Official capital investment data from the National Bureau of Statistics of China (NBSC) are recorded by two main annual series, "total investment in fixed assets (TIFA)" (*"quanshehui guding zichan touzi"* in Chinese) and "newly increased fixed assets (NIFA)" ("*xinzeng guding zichan*" in Chinese). TIFA and/or NIFA are supposed to be the basis for the gross fixed capital formation (GFCF) item in the Chinese national accounts. However, these indicators do not appear to be consistent (Figure S5), hence causing confusions to their users.

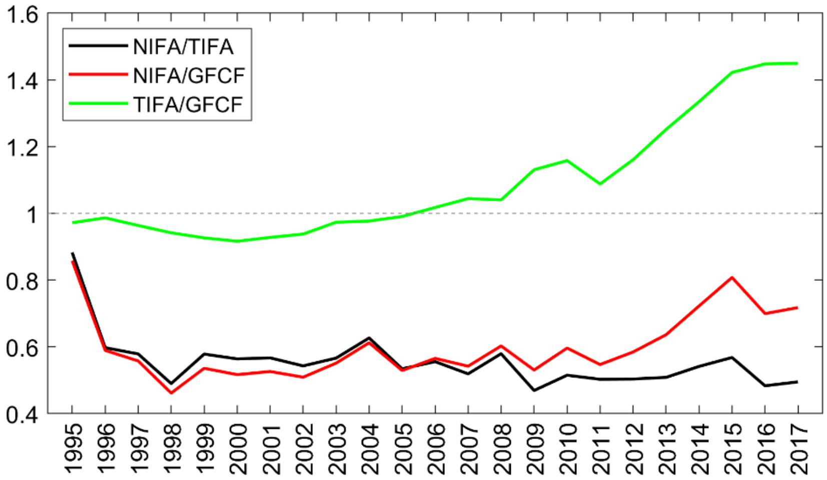

Figure S5. Relationship between Chinese statistical capital data. TIFA=total investment in fixed assets; NIFA=newly increased fixed assets; GFCF=gross fixed capital formation. Data source: National Bureau of Statistics of China.

An often made, significant mistake is the direct use of TIFA as the investment variable in estimating capital stock or capital depreciation with the perpetual inventory method (PIM, details see section **SI 7**) [22-26], which is conceptually inappropriate. By official definition, TIFA refers to the "workload" of activities in construction and purchases of fixed assets in money terms [15], which may not produce results that meet standards for fixed assets in the current period or may take many years to become qualified for fixed assets and some may never meet the standards, hence be completely wasted, which is a typical phenomenon in all centrally planned economies [27]. The problem is aggravated in the case of a large project because its investment "workload" is counted by stage of construction, but it cannot be used for production (hence should be counted as the increase in inventory) before all stages are completed and the operation actually commences. It can be sure that the official TIFA indicator and hence GFCF exaggerates the real level of fixed asset investment.

Compared with TIFA, the series of NIFA is much more compatible with the concept of "fixed asset investment" used in PIM because it refers to the value of investment projects completed and put into production or meeting the standards for fixed assets in the current year [15], hence reflecting the fixed assets formed in the current period as a results of those *effective* investment projects taking place in the current and previous periods. They are *effective* because they have been (or will be) turned into new fixed assets for production services rather than wasted.

If denote NIFA as N (in Yuan per year) and TIFA as M (or the "workload" of investment projects, in Yuan per year), assuming no coverage problem and double counting, then N in period t is the sum of M's in τ+1 periods (i=0, 1, 2, …, τ) multiplied by their respective ratios Θ (Θ<1), defined as, in value terms, the proportion of actually completed investment in period t in the total "workload" of the investment projects taking place in period t-I, that is,

$$N_t = \sum_{i=0}^{\tau} \Theta_{t-i} M_{t-i}\,,\ (i=0, 1, 2, \ldots \tau)$$

It should be mentioned that there is little information available on Θ and τ. An officially often used ratio, namely "rate of fixed assets put into use" ("*guding zichan jiaofu shiyonglv*" in Chinese) defined as $N_t/M_t = \sum_{i=0}^{\tau} \Theta_{t-i} M_{t-i} / M_t$, is misleading because it compares two concepts that are virtually incompatible (see Table 10-17 in China Statistical Yearbook 2017 [15]).

Although NIFA (denoted as N) is more reasonable than TIFA to be used as capital investment (denoted as I, in Yuan per year) in PIM, an upward adjustment has to be made to transfer N to I. This upward adjustment is to include the projects less than half million Yuan by non-state firms that are not reported in official investment statistics plus the value of likely underreported[28]. The standard I by sector s of province m in year t could be estimated as:

$$I_{m,t,s} = \frac{N_{m,t,s}}{1-\lambda_{m,t,s}},\ (\lambda<1)$$

where λ is to adjust N by the effects of missing and/or underreported investment. There is little information available on λ especially those at provincial level. We apply the national $\lambda_{t,s}$ from Wu [29] to adjust $N_{m,t,s}$, and further scale $N_{m,t,s}$ into the national capital investment by sector s in year t from WORLDKLEMS[9].

There are limited investment data by asset type especially at industrial level. In the official investment statistics, under the subcategories of TIFA 'capital construction' and 'technical update and transformation', there are data for 'equipment' and 'structures'. The 'structures' indicator also distinguishes 'housing' or 'non-productive' constructions. We rely on TIFA by these categories (although they are not directly relevant with NIFA), and industrial investment statistics in annual statistics bulletins[30] about industry and transportation economy, commune and brigade factories, and township and village enterprises to disaggregate the capital investment. According to Wu[29], this study also disaggregates four categories of industry-specific fixed assets, namely, 'equipment', 'residential structures', 'non-residential structures' and 'others'. We re-allocate 'others' into 'equipment' and 'non-residential structures' by a ratio of 3:7 according to Wu[29]. Without category-specific data on investments in non-industrial sectors (i.e., agriculture, construction, and all services), we assume that the non-industrial sector-specific I is equal to the official NIFA of that sector. We use the share of productive structures given by the economic-wide TIFA to decompose the total investment into non-residential structures and equipment.

A remaining question is how significant the "waste" part of capital assets (i.e., *non-effective* investment) would contribute to economic production and consumption, and further to the emissions of China. Given that there is no specific data or percentage of *non-effective* capital assets in total TIFA or NIFA, we rely on other data sources to estimate the contribution from waste. We take construction sector as an example, which accounted for 58% of total gross fixed capital formation (GFCF) of China during the period of 1995-2017 (see the main text). We obtain the construction waste data from Stadler et al. (2018)[31] for the year 2016, and assume all the construction waste are capital asset waste. This assumption must overestimate the amount of capital asset waste because there are other waste generated in construction work but not counted as capital assets such as ashes. We find that construction waste accounted for 2.5% of total GFCF of construction, and 1.6% of total supply chain-wide CO_2 emissions embodied in. Considering that these figures are overestimated, we conclude the waste capital assets are insignificant in total build-up capital in China.

7. The Perpetual Inventory Method (PIM)

As stated by European System of Accounts (ESA, 1995)[25], consumption of fixed capital should be estimated based on a gross capital stock and average lifespans of different types of capital assets. The Perpetual Inventory Method (PIM) is advised to estimate gross fixed capital stock. In this Box the basic principles of the PIM are discussed.

Investment series. Full implementation of the PIM requires relatively long time series of gross fixed capital formation (GFCF), broken down by type of fixed assets and institutional sectors. Such a data set is pre-constructed for 31 provinces of China in this study, as described in the section **SI 6**.

Depending on the economic structure of the country under consideration, certain types of assets may be important to be singled out in addition. For example, in some developing countries, cultivated assets such as livestock for breeding may be an important type of productive capital. In economies that are resource-rich, subsoil assets such as coal, oil or mineral reserves or non-cultivated biological resources such as natural forests may play an important role. General classifications of fixed capital applied in EU-KLMES (https://euklems.eu/) include transport equipment, ICT equipment, dwellings, computer software and databases.

Calculation of net capital stocks. The computational approach towards the measurement of capital depreciation and net capital stocks is by using a constant, age-independent rate of consumption of fixed capital (i.e., geometric rate or depreciation rate). This (simplified) practice dispenses from the need to specify extra parameters for a retirement profile and it permits to formulate a straight forward link between capital investment, capital stock, and consumption of fixed capital:

$$S_{tE} = S_{tB} + I_t - \delta(I_t + S_{tB}) + X_t$$

where S_{tE} and S_{tB} are the end-year and beginning-of-the year net capital stocks, I_t is gross fixed capital formation in year t, $\delta(I_t+S_{tB})$ is consumption of fixed capital in year t, and X_t is other changes in volumes of the group of assets. All variables are valued at average prices of a reference period which could be year t.

Depreciation rates. Computing the net stock above requires a rate of consumption of fixed capital, δ. Absent good information about the rates of depreciation, δ can be set by reference to other countries' depreciation rates of similar types of assets or other countries' lifespans of similar types of assets. A common way of estimating δ is the declining balance method with $\delta=R/T_a$ where T_a is the average lifespans of an asset a, and R is a parameter around 2 [26]. Because lifespans tend to be influenced by institutional and climatic conditions, it is preferable to use parameters from similar countries rather than from very different countries. EU-KLEMS and WORLDKLEMS provide detailed depreciation rate of different types of capital assets that are used by different economic sectors.

Consumption of fixed capital. Consumption of fixed capital is the amount of fixed assets used up during the period under consideration. More plain understanding of consumption of fixed capital is the deduction of gross capital stock. Reasons for the deduction are normal wear and tear and foreseeable obsolescence, including a provision for losses of fixed assets as a result of accidental damage which can be assured against. Based on the capital stock calculated by PIM, the consumption of fixed capital can be calculated as $\delta(I_t+S_{tB})$.

8. Constructing China's inter-provincial MRIO table series (1995-2017)

The basic framework to construct China's inter-provincial MRIO table series (1995-2017) follows previous studies [32-35], and uses the GRAS method [36]. The GRAS method is a branch of the RAS method, which is a procedure that is widely used for updating IO information over time. Here we present a brief introduction. We denote the column sum of the intermediate input matrix **Z** as **U**, while denote the row sum of **Z** as **V**. The RAS method is used to quantify the intermediate input matrix **Z**' in the target year, given the matrix **Z** in the reference year and **U**', **V**' and **x**' in the target year. The quantification procedure is to give iterative trial and adjustment of **U**' and **V**' to obtain a balanced matrix **Z**'. The units of all the elements in Chinese MRIO tables are Yuan.

The current best available MRIO tables in 2007 [10], 2010 [11], 2012 [12], 2015 and 2017 from CEADs [13], 1995-2006 from Wang [14] are relied on as MRIO tables in the reference years. Although Wang [14] construct the time series of MRIO tables since 1978, the features of these MRIO tables are quite different from the statistical data recorded in the National Bureau of Statistics of China (NBSC). An example of provincial value added in 2012 recorded in the NBSC, and from MRIO tables from Liu et al. [12] and Wang [14], respectively, are listed in Table S5. Because of the big differences of data in the MRIO tables complied by Wang [14] from those in the NBSC, we only use the MRIO tables of the period 1995-2006 from Wang [14] for our analysis. Besides, before we construct the MRIO tables in the missing years, we first adjust the final demand, exports, imports and value-added data in the existing MRIO tables, to make sure all the data compiled in MRIO tables are well balanced with the statistical data from the National Bureau of Statistics of China. The intermediate input table is then adjusted using the GRAS method to make sure the balances between total outputs and total inputs.

Table S5. Provincial value added in 2012 recorded in the National Bureau of Statistics of China (NBSC), MRIO tables from Liu et al. [12] and Wang [14], respectively. Unit in 10^8 Yuan.

Provinces	NBSC	Liu et al. [12]	Wang [14]
Beijing	19025	17879	6151
Tianjin	9043	12894	2226
Hebei	23077	26575	14924
Shanxi	11683	12113	6152
Inner Mongolia	10470	16372	4346
Liaoning	17849	24898	9108
Jilin	8678	11939	4213
Heilongjiang	11016	13733	6173
Shanghai	21306	20184	7326
Jiangsu	53702	59972	21799
Zhejiang	34382	35911	19614
Anhui	18342	17214	9694
Fujian	20191	19702	8377
Jiangxi	12808	12949	7774
Shandong	42957	50028	20624
Henan	28962	29599	15031
Hubei	22591	22415	9650
Hunan	21207	22154	10855
Guangdong	57008	55463	29217
Guangxi	11304	13035	5469
Hainan	2789	2856	1067
Chongqing	11595	11410	4450
Sichuan	23922	23873	13848
Guizhou	6742	6852	3960

Yunnan	11097	10371	6125
Tibet	710	701	221
Shaanxi	14142	14454	6518
Gansu	5393	5658	3149
Qinghai	1528	1894	813
Ningxia	2131	2347	1050
Xinjiang	7412	7509	3489

It should also be noted that the MRIO tables in 2007 and 2010 only have 30 regions (without Tibet) and 30 sectors, while the MRIO tables in 1995-2006, 2012, 2015 and 2017 have 31 regions and 42 sectors. To ensure the consistency of MRIO table time series, we omit all the transactions relevant to Tibet in the MRIO tables in 2012, 2015 and 2017, and disaggregate the 30 sectors into 42 sectors (Table S6) for further calculation. We also specify five final demand categories, i.e., final expenditures of rural population, final expenditures of urban population, final expenditures of government, gross fixed capital formation (GFCF), and stock changes, according to the best available MRIO tables.

Table S6. List of the 42 sectors in China's inter-provincial MRIO table time series.

Sector Number	Full Name	Short Name
1	Agriculture, forestry, animal husbandry and fishery products and services	Agri. sect.
2	Coal mining products	Coal mining
3	Oil and natural gas extraction products	Oil and nat. gas
4	Metal ore mining and products	Metal ore mining
5	Non-metallic minerals and other mining products	Mineral mining
6	Food manufacturing and tobacco	Food & tobacco
7	Textile and products	Textile
8	Leather and down of textiles, clothing, shoes, hats and articles thereof	Leather n.e.c
9	Wood products and furniture	Wood mfg.
10	Paper printing, culture, education, and sporting goods	Paper n.e.c
11	Petroleum, coking products and nuclear fuel processed products	Petroleum n.e.c
12	Chemical product	Chemical prod.
13	Non-metallic mineral product manufacturing	Mineral prod.
14	Metal smelting and rolling product manufacturing	Metal smelting
15	Metal product manufacturing	Metal prod.
16	General equipment	General eq.
17	Professional equipment	Professional eq.
18	Transportation equipment	Transportation eq.
19	Electrical machinery and equipment	Electricity eq.
20	Communication equipment, computers and other electronic equipment	Electronic eq.
21	Instrumentation	Instrumentation
22	Other manufactured products	Other mfg.
23	Waste of materials	Waste of materials
24	Repair of metal products, machinery and equipment	Repair mfg
25	Production and supply of electricity and heat	Electricity supply
26	Gas production and supply	Gas supply
27	Water production and supply	Water supply

28	Construction	Construction
29	Wholesale and retail	Wholesale etc.
30	Transportation, storage and post services	Transport. sev.
31	Accommodation and restaurant	Accommodation
32	Information transfer, software and information technology services	Info. sev.
33	Financial services	Financial sev.
34	Real estate services	Real estate
35	Leasing and business services	Business sev.
36	Scientific research and technical services	Sci. res. tech.
37	Public services, hydrology, environment and public facilities management	Public sev.
38	Resident services, repairs and other services	Resident sev.
39	Education	Education
40	Health and social work	Health sev.
41	Culture, sports and entertainment	Culture sev.
42	Public administration, social security and social organization	Public admin. sev.

To estimate **U**′, **V**′ and **x**′ in the target year, we first estimate final demand **y**′ and the export **EX**′ in the target year, and then we assume **U**′, **V**′ and **x**′ will all change proportionally with total changes in **y**′ and **EX**′. Final expenditures of rural population ($\mathbf{y\_r}'_m$) and final expenditures of urban population ($\mathbf{y\_u}'_m$) in the target year of province *m* are estimated in this way: we first determine the changes in product-specific expenditures of rural population (also for urban population) in the target year of province *m* from that in the reference year, which are calculated using the statistical data of product-specific per-capita expenditures of rural population (also for urban population) of province *m*, the number of rural population (also for urban population) of province *m*, and the inflation rates of associated years of province *m*; then we apply the product-specific changes to the final expenditures of associated producing sectors in $\mathbf{y\_r}_m$ (also for $\mathbf{y\_u}_m$); finally we balance $\mathbf{y\_r}'_m$ (also for $\mathbf{y\_u}'_m$) into the statistical data of gross final expenditures of rural population (also for urban population) in the target year of province *m*. Since there is no detailed per-capital expenditure data of government expenditures (**y_g**), we rely on the changes in final expenditures of rural and urban population in the target year from that in the reference year to estimate $\mathbf{y\_g}'_m$ in the target year, and also balance it into the statistical data of gross final expenditures of government in the target year of province *m*. GFCF ($\mathbf{y\_gfcf}'_m$) and stock changes ($\mathbf{y\_s}'_m$) in the target year of province *m* are estimated in the similar way of $\mathbf{y\_r}'_m$ or $\mathbf{y\_u}'_m$, but both rely on the changes in newly constructed capital investment time series, and finally balance them into the statistical data of gross GFCF and gross stock changes in the target year of province *m*, respectively. It should be noted that the statistical data of gross final expenditures of rural population, urban population, and government, GFCF, and stock changes of each province include the imported part. Therefore, when we estimate **y**′ in the target year, we distinguish **y**′ into domestic and imported ones. Since we only have nationally product-specific export data, we first determine product-specific export changes in the target year from the reference year, and proportionally adjust the export of each province based on the national changes in associated producing sectors, and lastly balance **EX**′ into the nationally product-specific export data in the target year. We believe relying on more actual statistical data will reduce the uncertainty in estimating **y**′ and **EX**′ as much as possible.

9. Constructing capital consumption time series

The procedures to trace and allocate the contribution of year *t*'s capital investment to year *n*'s inter-industrial production networks are referred to the global capital endogenized MRIO model[3]. The key step to obtain the supply chain-wide capital consumption matrix $\mathbf{D}^K_{t,n}$ ($t \leq n$, in Yuan) within China is re-creating the concordance tables that are used to convert capital assets and capital consumption sectors (37 sectors, Table S3) into the sectoral classifications of MRIO tables (42 sectors, Table S6). Details about how to trace and allocate the contribution of year *t*'s capital investments to year *n*'s inter-industry production networks depicted by year *n*'s MRIO tables, obtaining $\mathbf{D}^K_{t,n}$ ($t \leq n$) are described below.

The process to construct $\mathbf{D}^K_{t,n}$ is composed of five segments. First, annual capital consumption from the capital investment times series is calculated. We modeled consumption in year *n* of asset *a* invested in year *t* by sector *s* in province *m* as capital depreciation ($D^K_{m,a,s,t,n}$) calculated using the geometric method (**Eq. 9-1**). Geometric depreciation depicts each year the asset is depreciated by a constant percentage of the previous periods value (see the section **SI 7**).

$$D^K_{m,a,s,t,n} = \delta^K_{a,s}\left(1-\delta^K_{a,s}\right)^{n-t} I^K_{m,a,s,t} \qquad (9\text{-}1)$$

Capital investment values ($I^K_{m,a,s,t}$) are from our newly developed dataset (see the sections **SI 5** and **6**). Corresponding depreciation rates ($\delta^K_{a,s}$) are obtained from WORLDKLEMS[37]. δ are constant throughout the years of our simulation, but vary by asset *a*, the capital investing sector *s*. Note, we assume that the capital investing sectors are also the capital consuming sectors. Mathematically, $\mathbf{D}^K_{m,t,n}$ is a two-dimension matrix variable with the same shape as $\mathbf{I}^K_{m,t}$: sectors that invested and consumed assets are aligned by columns, assets are aligned by rows, while each element in $\mathbf{D}^K_{m,t,n}$ is calculated through Eq. 9-1.

The second step is to link capital consuming sector *s* to capital producing sector *s** through capital asset *a*. Each element in the transformed $\mathbf{D}^K_{m,t,n}$ matrix is $\mathbf{D}^K_{m,s^*,s,t,n}$. Such a transformation is achieved through 'asset-capital producing sector' concordance tables created in this study.

We then further distinguish the capital producing sectors to those located in province *m* and those outside of province *m*, i.e., capital assets that were inter-provincially imported. Such allocation is based on year *t*'s fixed capital formation matrix $\mathbf{Y}^K_{m,t}$ (in Yuan) from the inter-provincial MRIO tables. $\mathbf{Y}^K_{m,t}$ presents province *m*'s investment records in year *t*, specifying the expenditures across 42 sectors and 31 provinces. This step transforms the $\mathbf{D}^K_{m,t,n}$ matrix again, expanding the number of rows (producing sectors) from 42 to 1302 (42×31).

Next, we map the capital consuming sectors *s* that are defined in the macroeconomic datasets (e.g., 37 sectors in WORLDKLEMS) to 42 MRIO sectors. Such a transformation is also achieved through 'asset-capital producing sector' concordance tables created in this study. This step transforms $\mathbf{D}^K_{m,t,n}$ to a matrix with 1302 rows specifying capital production across China and 42 columns specifying capital consuming sectors in province *m*. The transformation does not change the sum of all elements in $\mathbf{D}^K_{m,t,n}$.

The final step in creating the capital consumption matrix $\mathbf{D}^K_{t,n}$ is to horizontally concatenate the aforementioned developed $\mathbf{D}^K_{1,t,n}$, $\mathbf{D}^K_{2,t,n}$, …, $\mathbf{D}^K_{31,t,n}$ for each of the 31 provinces. $\mathbf{D}^K_{t,n}$ is thus a 1302×1302 matrix with capital producing and capital consuming sectors along rows and columns, respectively; each element records the quantity of assets that were invested in year *t* and consumed (i.e., depreciated) in year *n*.

10. Summary of the 'business-as-usual' (BAU) scenario and the two capital investment scenarios

Table S7. Summary of the 'business-as-usual' (BAU) scenario and the two capital investment scenarios. A is the direct coefficient matrix showing inputs per-unit total output. **vd** is the value-added matrix. $\mathbf{Y}^{FC}$ is the final consumption matrix. $\mathbf{Y}^{GFCF}$ is the gross fixed capital formation (GFCF) matrix. **F** is a row vector of direct CO_2 emissions of economic activities.

	**Scenarios**		
	BAU	KES	KLC
Explanation	Economic development (including capital investment) and CO_2 emissions following current paths and climate policies	A particular increase in infrastructure investment (i.e., transportation, power, water, and communication) to improve economic growth and social well-being*	A particular increase in capital investment in low-carbon technologies by electricity generation sector, and end-use sectors such as transportation services**
Changes in **A**	General reductions in direct inputs due to the improvement in production efficiency, and particular changes in energy related sectors due to the changes in energy mix	Same as BAU	Adjusting **A** in BAU according to the energy mix changes under the low-carbon development
Changes in $\mathbf{Y}^{FC}$	Estimated by the predicted population, and per-capita final expenditure	Particular increase in $\mathbf{Y}^{FC}$ from the seven infrastructure-related sectors compared with BAU	Particular increase in $\mathbf{Y}^{FC}$ from the using sectors of low-carbon technologies compared with BAU
Changes in $\mathbf{Y}^{GFCF}$	Estimated by the capital investment of each investing sectors, and the capital production structure in the base year	Allocating specific investment in seven infrastructure categories to associated capital producing sectors, and further adjusted into the capital intensity of unit GDP in BAU	Allocating specific investment in low-carbon technologies by electricity generation sector and end-use sectors to associated capital producing sectors, and further adjusted into the capital intensity of unit GDP in BAU
Changes in **vd**	GDP growth rate set as 6.5% before 2020, and 5% after 2020	Changes according to $\mathbf{Y}^{FC}$ and $\mathbf{Y}^{GFCF}$	Changes according to $\mathbf{Y}^{FC}$ and $\mathbf{Y}^{GFCF}$
Changes in **F**	Consistent with the change in the intermediate inputs	Consistent with the change in the intermediate inputs	Adjusting **F** in BAU according to the CO_2 emissions of generation sector and end-use sectors under the low-carbon development

Notes: * associated capital investment in each infrastructure are collected from Global Infrastructure Hub [38]; ** associated capital investment in low-carbon technologies by electricity generation sector and end-use sectors are collected from World Energy Outlook [39], and only relative changes in key parameters such as CO_2 intensity of one-unit GDP are used.

11. Major parameters in the three capital investment scenarios.

Table S8. Major parameters in the three capital investment scenarios.

	Base year (2017)*	BAU (2030)	KES (2030)	KLC (2030)
Economic-related				
GDP (in billion, 2017 Yuan)	83210	163725	165556	156905
Share of GFCF in GDP	42.7%	42.7%	42.4%	46.6%
Electricity price (annual change until 2030)		0.93%		
Cumulative investment (in billion, 2017 Yuan)**				
Electricity/water		28656	31327	
Transportation		47402	51731	
Telecommunication		3543	3750	
Low-carbon technologies				13003
*Social-related****				
Population (billion)	1.39	1.46		1.45
Urbanization rate	59.0%	70.8%		71.6%
*Energy-mix-related*****				
Total energy supply (million toe)	2490	3540	3578	2820
Coal	1819	2353	2379	1802
Oil	195	252	255	156
Natural gas	122	213	215	191
Nuclear	65	278	281	218
Renewable	289	442	447	453
Power generation (TWh)	6557	9321	9426	8771
Total energy use (million toe)				
Coal	1970	2549	2577	1873
Oil	578	822	831	711
Gas	198	393	397	374
Nuclear	65	164	166	218
Renewables	289	475	480	455

Note: * data for the base year were collected from the National Bureau of Statistics of China [7]; ** the cumulative investment in the seven infrastructure under the KES, and on the low-carbon technology under the KLC are listed in this table, which do not show the total cumulative investment from all 42 economic sectors; *** future population and urbanization rate of China are collected from Chen et al. [40]; **** the associated energy mix data of China are collected from the New Policy Scenario developed in the World Energy Outlook 2017 [39].

12. Applying energy mix changes in MRIO tables

We follow the method proposed by Wiebe et al. (2018)[41] to apply the energy mix changes in the MRIO tables. The sketch of procedures to apply energy mix is shown in Figure S6. Data related to energy supply and use by sectors are listed in Table S8.

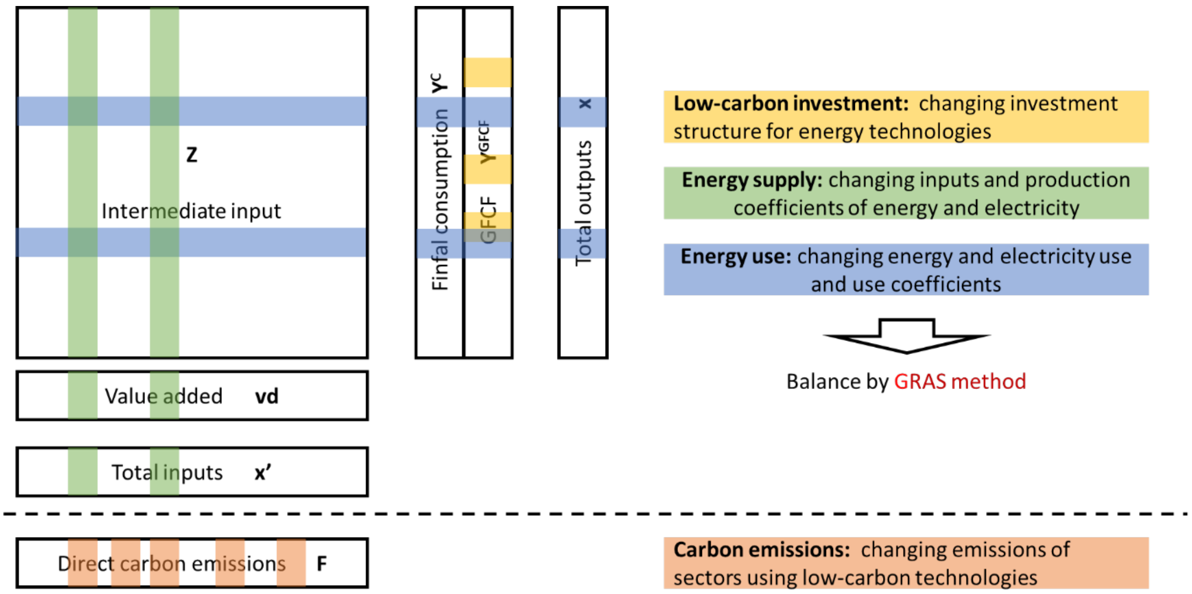

Figure S6. Diagram to apply energy mix changes in MRIO tables.

13. Relationships between sectorial capital investment and final consumption

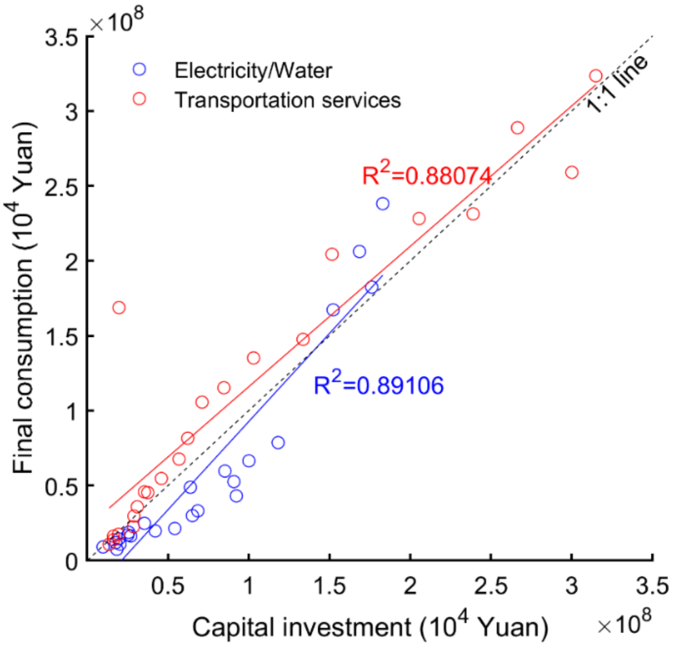

Figure S7. Trends in capital investment by, and final consumption of electricity/water production and supply sector as well as transportation service sector.

Each scatter in this plot represents the pair of capital investment and final consumption of associated sector in one year. The data sources of capital investment and final consumption could be found in the section **SI 5**.

14. Changes in per-capital CO_2 emissions

The following table is used to support Figure 2b in the main text.

Table S9. Changes in regional per-capita PBEs and CBEs for the year 2017 with and without the re-allocation of F^K. The units of per-capita PBE, CBE, PBE^K, and CBE^K are tonnes.

	Production-based			Consumption-based			
**Regions**	PBE	PBE^K	Relative Changes (PBE^K to PBE)	CBE	CBE^K	Relative Changes (CBE^K to CBE)	Relative Changes (CBE to PBE)
Beijing-Tianjin	3.9	3.3	-15%	5.8	4.1	-30%	48%
Northeast	6.1	4.0	-34%	4.5	3.3	-28%	-26%
North	6.2	3.9	-37%	5.5	3.8	-30%	-12%
Central Coast	6.3	4.9	-22%	7.2	5.4	-26%	15%
South Coast	3.8	2.7	-27%	4.0	3.0	-26%	7%
Southwest	3.7	2.5	-31%	4.2	2.7	-36%	15%
Central	4.1	3.0	-27%	5.3	3.4	-36%	30%
Northwest	11.5	7.2	-38%	7.1	5.2	-26%	-39%

15. The logic of temporal allocation of capital-associated CO_2 emissions to production-based emissions

We did realize that our re-allocation method to production-based emissions (PBE) may cause some confusion or unclearness from the conventional definition of "production-based accounting"[42,43]. Conventional PBE of a country is defined as CO_2 occurring at the production sites within a national territory. This definition is based on the logic that emissions are assigned to the place of economic production and value added creation. A similar logic also applies to consumption-based emissions—emissions (accruing throughout global value chains) are assigned to the place of final consumption.

We can ensure that our re-allocation method to PBE follows the conventional logic of production-based emission assignment. The main reasons for the confusion lie in the unique features of capital assets. Different from non-capital goods, capital assets undergo depreciation processes.

We take production, purchase, and use of electricity generators (representing capital assets) and coal (representing non-capital goods) as examples. We assume that electricity generators and coal are produced in China (CHN), and then purchased by the United States (USA) for USA's economic production. The table below shows to which country value added and CO_2 emissions of electricity generators and coal should be assigned.

	**Assigning to which country?**	
	Value added (notes)	CO_2 emissions (notes)
Production of		
electricity generators	CHN (as GDP of)	CHN (as PBE of)
coals	CHN (as GDP of)	CHN (as PBE of)
Depreciation processes of		
electricity generators	USA (counted as consumption of fixed capital, as GDP of)	USA (counted as F^K, as PBE^K of)
coals	—	—
Economic production using		
electricity generators	USA (as GDP of)	USA (as PBE of)
coals	USA (as GDP of)	USA (as PBE of)

From the table, we can see capital assets undergo depreciation processes, which also generate value-added. The generated value added due to capital depreciation (i.e., consumption of fixed capital) is accounted as part of GDP of the capital purchasing country (in the above case, the USA). As such, under the logic of conventional production-based accounting, we should allocate the CO_2 emissions embodied in capital depreciation (counted as F^K of PBE^K) to the purchasing country, wherever the embodied CO_2 occurs.

In addition, at a global scope, PBE should be equal to CBE. Under the accounting framework of our study, this relationship is spread over time to when the use (as measured by depreciated capital) occurs. The equation still holds but with the additional time dimension.

SI References

1 Lenzen, M. & Treloar, G. J. Endogenising Capital A comparison of Two Methods. *J. Appl. Input-Output Anal.* **10**, 1-11 (2004).

2 Chen, Z.-M. *et al.* Consumption-based greenhouse gas emissions accounting with capital stock change highlights dynamics of fast-developing countries. *Nature communications* **9**, 3581 (2018).

3 Ye, Q. *et al.* Linking the Environmental Pressures of China's Capital Development to Global Final Consumption of the Past Decades and into the Future. *Environ Sci Technol* **55**, 6421-6429, doi:10.1021/acs.est.0c07263 (2021).

4 Södersten, C.-J. H., Wood, R. & Hertwich, E. G. Endogenizing capital in MRIO models: the implications for consumption-based accounting. *Environ Sci Technol* **52**, 13250-13259 (2018).

5 Södersten, C.-J., Wood, R. & Wiedmann, T. The capital load of global material footprints. *Resources, Conservation and Recycling* **158**, 104811 (2020).

6 Södersten, C.-J. H., Wood, R. & Hertwich, E. G. Environmental Impacts of Capital Formation. *Journal of Industrial Ecology* **22**, 55-67, doi:10.1111/jiec.12532 (2018).

7 NBSC. Annual Statistics Data by Province (accessed on April 06, 2020). *National Bureau of Statistics of China (NBSC)* (2020).

8 NBSC. Statistical Yearbook of the Chinese Investment in Fixed Assets. *Fixed Assets Investment Statistics Department of National Bureau of Statistics*. China Statistics Press, Beijing (2018).

9 WORLDKLEMS. *WORLD KLEMS Initiative (accessed on Oct. 17, 2019)*, <http://www.worldklems.net/index.htm> (2019).

10 Liu, W. D., T, Z. & Chen, J. The Multi-Regional Input-Output Table of 30 Regions in China in 2007. (2012).

11 Liu, W. D., T, Z. & Chen, J. The Multi-Regional Input-Output Table of 30 Regions in China in 2010. (2014).

12 Liu, W. D., T, Z. & Chen, J. The Multi-Regional Input-Output Table of 31 Regions in China in 2012. (2018).

13Zheng, H. *et al.* Regional determinants of China's consumption-based emissions in the economic transition. *Environmental Research Letters* **15**, doi:10.1088/1748-9326/ab794f (2020).

14 Wang, Y. An industrial ecology virtual framework for policy making in China. *Economic Systems Research* **29**, 252-274, doi:10.1080/09535314.2017.1313199 (2017).

15 NBSC. Statistic Yearbook of the People's Republic of China. *National Bureau of Statistics of the People's Republic of China (NBSC)*, Beijing, China (2017).

16 NBSC. CHINA RURAL STATISTICAL YEARBOOK. *National Bureau of Statistics Rural Social and Economic Investigation Department*. China Statistics Press, Beijing (2018).

17 NBSC. CHINA TRADE AND EXTERNAL ECONOMIC STATISTICAL YEARBOOK. *National Bureau of Statistics Trade and Foreign Economic Statistics Department.* China Statistics Press, Beijing (2017).

18 NBSC. MARKET STATISTICAL YEARBOOK OF CHINA. *National Bureau of Statistics Department of Foreign Trade Economic Planning.* China Statistics Press, Beijing (1997).

19 Shan, Y. *et al.* China CO2 emission accounts 1997-2015. *Sci Data* **5**, 170201, doi:10.1038/sdata.2017.201 (2018).

20 Shan, Y., Huang, Q., Guan, D. & Hubacek, K. China CO2 emission accounts 2016-2017. *Sci Data* **7**, 54, doi:10.1038/s41597-020-0393-y (2020).

21 Shan, Y. *et al.* New provincial CO2 emission inventories in China based on apparent energy consumption data and updated emission factors. *Applied Energy* **184**, 742-750, doi:10.1016/j.apenergy.2016.03.073 (2016).

22 Huang, Y., Ren, R. & Liu, X. Capital Stock Estimates in Chinese Manufacturing by Perpetual Inventory Approach. *China Economics Quarterly* **1**, 377-396 (in Chinese) (2002).

23 Hu, Z. F. & Khan, M. S. Why Is China Growing So Fast? *IMF Staff Papers* **44**, 103-131 (1997).

24 Li, J. Productivity and China's Economic Growth. *The Economic Studies Quarterly* **43**, 337-350 (1992).

25 Eurostat. European System of Accounts ESA 1995. (1995).

26 OECD. Measuring Capital OECD Manual 2009 Second Edition. (2009).

27 Chow, G. C. Capital Formation and Economic Growth in China. *The Quarterly Journal of Economics* **108**, 809-842 (1993).

28 Young, A. Gold into Base Metals: Productivity Growth in the People's Republic of China during the Reform Period. *NBER Working Paper Series, w7856, National Bureau of Economic Research, Cambridge, MA* (2000).

29 Wu, H. X. Constructing China's Net Capital and Measuring Capital Services in China, 1980-2010. (2015).

30 DITS. Annual Statistical Bulletin on Industry and Transportation Economy (*Gongye Jiaotong Tongji Nianbao*)/Annual Statistical Bulletin on Commune and Brigade Factories (*Shedui Qiye Tongji Nianbao*)/Annual Statistical Bulletin on Township and Village Enterprises (*Xiangzhen Qiye Tongji Nianbao*). (multiple years).

31 Stadler, K. *et al.* EXIOBASE 3: Developing a time series of detailed environmentally extended multi-regional input-output tables. *Journal of Industrial Ecology* **22**, 502-515 (2018).

32 Guan, D., Hubacek, K., Weber, C. L., Peters, G. P. & Reiner, D. M. The drivers of Chinese CO2 emissions from 1980 to 2030. *Global Environmental Change* **18**, 626-634, doi:10.1016/j.gloenvcha.2008.08.001 (2008).

33 Hubacek, K. & Sun, L. A scenario analysis of China's land use and land cover change incorporating biophysical information into input–output modeling. *Struct Change Econ Dynam* **12**, 367-397 (2001).

34 Hubacek, K. & Sun, L. Economic and Societal Changes in China and their Effects on Water Use: A Scenario Analysis. *J Ind Ecol* **9**, 187-200 (2005).

35 Zhao, X. *et al.* Physical and virtual water transfers for regional water stress alleviation in China. *P Natl Acad Sci USA* **112**, 1031-1035, doi:10.1073/pnas.1404130112 (2015).

36Günlük-Şenesen, G. & Bates, J. M. Some experiments with methods of adjusting unbalanced data matrices. *Journal of the Royal Statistical Society* **Series A (Statistics in Society)**, 473-490 (1988).

37 WORLDKLEMS. *WORLD KLEMS Initiative*, <http://www.worldklems.net/index.htm> (accessed on Oct. 17, 2019).

38 Hub, G. I. Global infrastructure outlook: Infrastructure investment needs 50 countries, 7 sectors to 2040. (Sydney, Australia, 2017).

39 IEA. World Energy Outlook 2017. (International Energy Agency (IEA), Paris, 2017).

40 Chen, Y. *et al.* Provincial and gridded population projection for China under shared socioeconomic pathways from 2010 to 2100. *Sci Data* **7**, 83, doi:10.1038/s41597-020-0421-y (2020).

41 Wiebe, K. S., Bjelle, E. L., Többen, J. & Wood, R. Implementing exogenous scenarios in a global MRIO model for the estimation of future environmental footprints. *Journal of Economic Structures* **7**, doi:10.1186/s40008-018-0118-y (2018).

42 Peters, G. P. & Hertwich, E. G. Post-Kyoto greenhouse gas inventories: production versus consumption. *Climatic Change* **86**, 51-66, doi:10.1007/s10584-007-9280-1 (2007).

43 Peters, G. P. From production-based to consumption-based national emission inventories. *Ecological Economics* **65**, 13-23, doi:10.1016/j.ecolecon.2007.10.014 (2008).
